# Usefulness of Pathological Autopsy for Patients With Malignant Tumors

**DOI:** 10.7759/cureus.78114

**Published:** 2025-01-28

**Authors:** Yoshihiro Shibata, Tatsuhiro Kajitani, Atsushi Matsuyama, Ryuji Nakano

**Affiliations:** 1 Department of Oncology, Fukuoka Wajiro Hospital, Fukuoka, JPN; 2 Department of Pathology, Fukuoka Wajiro Hospital, Fukuoka, JPN

**Keywords:** autopsy, clinical diagnosis, discrepancy, goldman criteria, malignant tumor

## Abstract

Introduction

Pathological autopsy is useful for elucidating pathological conditions and causes of death that are not known clinically. In recent years, pathological autopsies have decreased worldwide, and few studies have evaluated the discrepancy between clinical diagnosis and autopsy findings of malignant tumors. Goldman criteria classify discrepancies between clinical and pathological autopsy diagnoses, as follows: Class I (major discrepancies), missed major diagnosis; Class II (obvious discrepancies), missed major diagnosis; Class III, missed minor diagnosis; and Class IV, other missed minor diagnoses.

Objective

The Goldman criteria are used to assess discordance between antemortem clinical diagnosis and pathological autopsy, with major discordance rates reported at 16.6%-59%. The objective of this study was to investigate the usefulness of pathological autopsy in patients with malignant tumors.

Methodology

The Goldman criteria were applied to evaluate discordance between the antemortem clinical diagnosis and the diagnosis at pathological autopsy in 25 patients with malignant tumors who underwent pathological autopsy at the Department of Oncology, Fukuoka Wajiro Hospital, from December 2015 to May 2023, to assess the utility of pathological autopsy in clinical practice retrospectively.

Results

Eight patients (32%) were Class I, four (16%) were Class II in accordance with the Goldman criteria, and 11 (44%) were Class I/II, which was considered a major discordance. Class I discordance was difficult to diagnose by imaging and comprised histological discordance, sepsis, pulmonary infarction, disseminated intravascular coagulation, aplastic anemia, and bone marrow carcinomatosis. The most common conditions that were difficult to diagnose by imaging were metastases to the gastrointestinal tract, biliary system, and pancreas, which were more frequently revealed at pathological autopsy.

Conclusions

Pathological autopsy is a valuable tool for understanding the pathology of diseases as it reveals significant discrepancies between antemortem diagnoses and autopsy findings, particularly regarding disease extent, histological diagnosis, and causes of death.

## Introduction

Autopsies can be classified into four main types: investigative autopsies, which aim to determine the cause of death; pathological autopsies, which focus on understanding the underlying disease pathology; judicial autopsies, conducted in cases of suspected criminal deaths; and administrative autopsies, performed when the cause of death is unknown. Recently, with developments in diagnostic imaging, the number of pathological autopsies has decreased worldwide. Very few studies have assessed the discrepancy between clinical diagnosis and autopsy findings in malignancies. The reported major discordance rate between the antemortem clinical diagnosis and the pathological autopsy diagnosis in patients with malignant tumors ranges from 16.6% to 59% [1-3]. In pathological autopsies, the Goldman criteria are commonly and widely used [4]. These criteria classify discrepancies between clinical and pathological autopsy diagnoses, as follows: Class I (major discrepancies), missed major diagnoses with potential adverse impact on survival that would have changed management; Class II (obvious discrepancies), missed major diagnoses with no potential impact on survival and that would not have changed the therapy; Class III, missed minor diagnoses related to terminal disease but not related to the cause of death; and Class IV, other missed minor diagnoses.

In this study, we evaluated the discrepancy between the antemortem clinical diagnosis and the diagnosis at pathological autopsy in patients with malignant tumors using the Goldman criteria to determine the utility of pathological autopsy.

## Materials and methods

Patients

This retrospective study involved patients with malignant tumors who underwent pathological autopsy at the Department of Oncology, Fukuoka Wajiro Hospital, from December 2015 to May 2023.

We collected the following data from the patient’s medical records: age, sex, malignancy neoplasm, histopathological diagnosis, hospital stay at the time of death, date of last computed tomography (CT) imaging before death, history of anticancer treatment, status of malignancy treatment at the time of death, and the presence of life-prolonging treatment. We also collected the following data: disease stage, metastasis location, and other noted lesions from the imaging reports. We collected the following data from the pathological autopsy reports: main and secondary lesions, lesions related to the cause of death, and histological types. Discrepancies between the antemortem clinical diagnosis and the pathological autopsy diagnosis were evaluated using the Goldman criteria (Table 1) [4]. Metastases identified at pathological autopsy were classified as Class III late-stage lesions. It is difficult to identify all lymph node metastases, and considering the clinical significance, lymph nodes above the diaphragm were grouped, with a separate group for lymph nodes below the diaphragm. Pleural dissemination and malignant pleural effusion were grouped as organs of the thoracic cavity, and peritoneal dissemination, diaphragmatic dissemination, and malignant ascites were grouped as organs of the abdominal cavity.

**Table 1:** The Goldman criteria are used to evaluate discrepancies between antemortem clinical diagnoses and pathological anatomy.

Class	Description	Example
I	Missed major diagnoses with potential adverse impacts on survival and that could have altered management	Myocardial infarction without cardiac arrest and undetected gastrointestinal bleeding requiring treatment
II	Missed major diagnoses with no potential impact on survival and that would not have changed therapy	Gastric perforation not requiring treatment; tumor spread to previously unknown vital organs
III	Missed minor diagnoses related to terminal disease but not related to the cause of death	Unknown arteriosclerosis and untreated old myocardial infarction
IV	Other missed minor diagnoses	Asymptomatic cholecystitis and goiter

We hypothesized that a shorter time between the final imaging diagnosis and the pathological autopsy would decrease the discordance rate between the antemortem clinical diagnosis and the pathological autopsy diagnosis. Therefore, the Mann-Whitney U test was used to evaluate the association between the discordance rate between the antemortem clinical diagnosis and the pathological autopsy diagnosis, and the number of days between the final imaging diagnosis and death.

## Results

During the study period, 25 patients with malignant tumors underwent pathological autopsy: 17 men and 8 women, with a median age of 66 years (range: 49-88). Table 2 shows the types of malignancies at the time of antemortem clinical diagnosis, and Table 3 shows the details of cancer treatment and life-prolonging treatment. Most patients had a history of anticancer therapy, had progressed to best supportive care, and had advanced disease. In some cases, cardiopulmonary resuscitation was performed in response to an unexpected sudden change.

**Table 2:** Types of cancer at the time of antemortem clinical diagnosis (n = 25).

Types of cancer	Number
Lung cancer	5
Gastric cancer	4
Pancreatic cancer	4
Colon cancer	3
Breast cancer	3
Esophageal cancer	2
Acute myeloid leukemia	1
Esophageal sarcoma	1
Renal cell carcinoma	1
Colonic neuroendocrine carcinoma	1

**Table 3:** Details of cancer treatment, life-prolonging interventions, length of hospital stay at the time of death, and the interval from the last CT imaging to the date of death. BSC, best supportive care; CT, computed tomography

History and type of anticancer treatment	n
Chemotherapy	17
Chemoradiotherapy	2
Hormone therapy	1
No treatment (BSC)	5
Cancer treatment status at death	
During treatment	5
End of treatment, BSC	11
Receiving treatment for complications and comorbidities	4
No treatment (BSC)	4
Pre-treatment	1
Life-prolonging treatment	6/25
Chest compressions	3
Endotracheal intubation	1
Both	2
Length of hospital stay (days), median (range)	9 (1-45)
Final CT, number of days before death, median (range)	9 (1-59)

Eight patients (32%) were classified as Class I in accordance with the Goldman criteria, 4 (16%) were classified as Class II, and 11 (44%) were classified as Class I/II, which was considered a major discordance (Table 4). Metastases identified at autopsy were classified as Class III late-stage lesions. Two cases were diagnosed as adenocarcinoma by antemortem clinical diagnosis but were diagnosed as mucoepidermoid carcinoma and adenoneuroendocrine carcinoma, respectively, by pathological autopsy. Another case was diagnosed as spindle cell carcinoma by pathological autopsy, although the antemortem diagnosis revealed only malignant cells, with no specific histological diagnosis. Antemortem biopsy pathology can be used to evaluate only a portion of a lesion; however, pathological autopsy allows examination of the entire lesion and may provide a more accurate histological diagnosis.

**Table 4:** Discrepancy rates according to the Goldman Criteria.

Class	n	%
I	8	32
II	4	16
III	23	92
IV	18	72

Other Class I conditions included two cases of sepsis, and one case each of septic lung infarction, disseminated intravascular coagulation, aplastic anemia, bone marrow carcinomatosis, and bronchopneumonia/abscess. These conditions were determined as causes of death that deviated from the clinical diagnosis. It was considered that the clinical diagnosis was difficult to make through imaging alone, due to the variety of pathologies present at the end of life in patients with malignant tumors.

In Class II, there were two cases of overlapping cancers. In one case, a pathological autopsy revealed large cell carcinoma of the lung, thyroid carcinoma, and prostate carcinoma in addition to the primary disease. In the other case, thyroid cancer was newly detected. Both patients had no clinical symptoms and were at disease stages that did not affect survival.

The median date of the last imaging (CT) was nine days (range: 1-59 days) before death. There was no association between Class I discordance or Class I/II discordance and the time from the last CT imaging to the date of death (*P *= 0.29 and *P* = 0.38, respectively) (Table 5). The close proximity of the date of death to the date of imaging may have made it difficult to accurately ascertain the cause of death because the imaging was not performed postmortem (autopsy imaging [Ai]). We also found no association between Class I or Class I/II discrepancies and length of hospital stay at the time of death (*P* = 0.46 and *P* = 0.23, respectively) (Table 6).

**Table 5:** Association between major discrepancies and the number of days from the last CT imaging to death. The date of death was calculated based on the date of the last CT scan as day 1. CT, computed tomography

		n	Days from the last CT imaging to the date of death	Mann-Whitney U test
Class I	Match	17	9 (1-59)	*P *= 0.29
	Mismatch	8	6.5 (2-23)	
Class I/II	Match both	14	8.5 (1-59)	*P *= 0.38
	One or both mismatch	11	9 (2-51)	

**Table 6:** Association between major discrepancies and length of hospital stay at the time of death.

		n	Days in the hospital	Mann-Whitney U test
Class I	Match	17	10 (1-35)	*P *= 0.46
	Mismatch	8	8.5 (4-45)	
Class I/II	Match both	14	9.5 (1-35)	*P *= 0.23
	One or both mismatch	11	9 (4-45)	

The most common metastatic sites identified at pathological autopsy were the lung (18/25, 72%), liver (16/25, 64%), supradiaphragmatic lymph nodes (15/25, 60%), subdiaphragmatic lymph nodes (14/25, 56%), adrenal glands (9/25, 36%), and abdominal cavity (9/25, 36%) (Table 7). Metastasis to the lungs and liver, where blood flow is abundant, was more common compared to other sites, and lymph node metastasis due to lymphatic spread was also frequently identified.

**Table 7:** Metastasis sites identified at pathological autopsy. Lymph nodes were grouped as lymph nodes above the diaphragm and lymph nodes below the diaphragm. Pleural dissemination and cancerous pleural effusion were considered organs of the thoracic cavity, and peritoneal dissemination, diaphragmatic dissemination, and cancerous ascites were considered organs of the abdominal cavity.

Metastasis site	n
Lung	18
Liver	16
Lymph nodes above the diaphragm	15
Lymph nodes below the diaphragm	14
Adrenal glands	9
Abdominal cavity	9
Colon	7
Stomach, pancreas, thoracic cavity	6
Kidney, spleen, small intestine	5
Esophagus, heart and pericardium, bladder, uterus, bone marrow, bone	4
Gallbladder and bile duct, ovary, cancerous lymphangiopathy	3
Spinal cord, trachea, subcutaneous tissues, prostate	1

A summary of the metastatic organs newly found by pathological autopsy (new cases diagnosed at autopsy/all cases found at autopsy), with their proportions, is as follows: esophagus (4/4, 100%), biliary system (3/3, 100%), stomach (5/6, 83%), pancreas (5/6, 83%), kidney (4/5, 80%), adrenal gland (7/9, 78%), heart and pericardium (3/4, 75%), colon (4/7, 57%), lung (10/18, 56%), and bone marrow (2/4, 50%) (Table 8). There was a trend toward more digestive tract lesions that were difficult to determine by CT imaging compared with other locations.

**Table 8:** Percentage of newly identified metastases at pathological autopsy. Values were calculated as the new cases diagnosed at autopsy/all cases found at autopsy.

Metastasis site	Newly identified rate, %
Esophagus	4/4, 100%
Gall bladder and bile duct	3/3, 100%
Trachea	1/1, 100%
Spinal cord	1/1, 100%
Stomach	5/6, 83%
Pancreas	5/6, 83%
Kidney	4/5, 80%
Adrenal glands	7/9, 78%
Heart and pericardium	3/4, 75%
Colon	4/7, 57%
Lung	10/18, 56%
Bone marrow	2/4, 50%
Spleen	2/5, 40%
Thoracic cavity	2/6, 33%
Cancerous lymphangiopathy	1/3, 33%
Bone	1/4, 25%
Lymph nodes above the diaphragm	3/14, 21%
Lymph nodes below the diaphragm	3/15, 20%
Small intestine	1/5, 20%
Liver	2/16, 16%
Abdominal cavity	1/9, 11%

## Discussion

The number (%) of pathological autopsy discrepancies in patients with malignancy in this study was 8 (32%) for Goldman criteria Class I and 4 (16%) for Class II. The number (%) for Class I/II, which is considered a major discrepancy, was 11 (44%), comparable to the results in previous reports [1-3]. Class I discrepancies, which are major discrepancies affecting survival, comprised histological discrepancies, sepsis, pulmonary infarction, disseminated intravascular coagulation, aplastic anemia, and bone marrow carcinomatosis, all of which are difficult to diagnose by imaging. We identified three cases in which the histological diagnosis was changed at pathological autopsy, and new concurrent cancers were identified in two cases. There was no association between the time from the last CT imaging to the date of death and the discordance rate. The most common metastasis sites identified at pathological autopsy were the lungs, livers, and lymph nodes. The most common newly identified metastasis sites at pathological autopsy were the esophagus, stomach, pancreas, and other gastrointestinal organs.

In Japan, the number of pathological autopsies peaked at 40,247 in 1985 and has been declining since; 10,019 autopsies were performed in 2019, which is one-fourth of the peak number [5]. In 1989, the autopsy rate at university hospitals was approximately 40% and that at general hospitals was approximately 20% [6]. However, in 2020, the rates were 4.9% and 1.9%, respectively, decreasing to a total of 2.4% [6]. The number of pathological autopsies at university hospitals and general hospitals that can perform pathological autopsies is reported in the Annual of Pathological Autopsy Cases in Japan. Although similar comparisons are difficult to make because reports from other countries do not distinguish between pathological autopsies and judicial or administrative autopsies, the decline in the rate of pathological autopsies is a worldwide trend. In the United States, the autopsy rate reached approximately 50% in the 1940s and has since declined; the reported autopsy rate for hospitalized deaths is now <1% [7]. In the United Kingdom, the reported autopsy rate for pathological autopsies was <1% in 2015 [8].

One reason for the decline in the autopsy rate may be that advances in diagnostic imaging and endoscopy have made it possible to obtain more information than previously available. Additionally, the increasing use of postmortem imaging (Ai) as a substitute for autopsy may be related to the fact that Ai may be useful to potentially determine antemortem conditions and the cause of death in cases related to judicial and administrative autopsies, such as trauma and hemorrhage. Pathological autopsies allow analysis of the histological type and spread of tumors as well as histological changes in degenerative diseases, which may be a limitation when considering the effectiveness of Ai.

The Goldman criteria [4] have been used to evaluate discordance between antemortem clinical and pathological autopsy diagnoses. A meta-analysis by Shojania et al. reported a Class I discordance rate of 9.0% (range: 0%-20.7%) and a major discordance rate of 23.5% (range: 4.1%-49.8%) for Class I and II combined [9]. The main discrepancy rate for Class I and II combined was 23.5% (range: 4.1%-49.8%). In the present study, the Class I discordance rate was 32% and that for Class I/II was 44%, comparable to results in previous reports [1-3]. Class I discrepancies in patients with malignant tumors have been reported to include pneumonia, sepsis, myocardial infarction, pulmonary embolism (PE), and gastrointestinal bleeding [1,3]. In the present study, Class I discordance for sepsis, PE, and disseminated intravascular coagulation, which were not seen in the clinical diagnoses, was also observed, and if the antemortem diagnosis had been possible, the treatment plans might have been changed. We also observed three cases of histological discordance, which may have changed the choice of therapeutic agents.

Concurrent cancers, although not involved in survival, were found in two cases, and the possibility of new and different tumors should always be considered in the clinical course of malignancy. de Pangher Manzini et al. reported that malignancy was found in 44% (457 cases) of 1,036 pathological autopsies [10]. Of these, 42% (228 cases) were not diagnosed before death.

In a review of pathological autopsies of 323 patients with lung cancer, a 16% discordance rate was found between antemortem cytology and histology and histological type at pathological autopsy [11]. Among the patients, the conformity rate of large cell carcinoma was low, at 54.8%, and most cancers were diagnosed as adenocarcinoma at pathological autopsy. Tsuji et al. compared biopsy and resection specimens of gastric tumors and reported that 12% of patients with two biopsies were not diagnosed with cancer [12]. The diagnosis rate did not improve with an increase in the number of biopsies. Comparing gastric endoscopic mucosal resection with biopsy diagnosis, Takao et al. reported that 17% of lesions diagnosed as undifferentiated by preoperative biopsy were found to be differentiated by postoperative pathology [13]. In the present study, histological discordance was confirmed in three cases. If the lesion cannot be excised antemortem and only biopsy is used for the pathological diagnosis, only a portion of the tissue can be diagnosed. In one case in the present study, the antemortem clinical diagnosis was adenocarcinoma, but the pathological autopsy revealed an adenoneuroendocrine carcinoma. The biopsy showed only an adenocarcinoma component, but the pathological autopsy expanded the evaluation area for the lesion and confirmed an endocrine carcinoma component. In another case, the antemortem diagnosis revealed only malignant cells, but the pathological autopsy confirmed the diagnosis of spindle cell carcinoma [14]. An extensive search of the lesion confirmed the presence of intraepithelial carcinoma and allowed for additional immunostaining studies, resulting in a definitive diagnosis. Pathological autopsy allows for the evaluation of the entire lesion, which increases the accuracy of the histological diagnosis.

The most common metastatic sites in the present study were the lungs, liver, lymph nodes, adrenal glands, and abdominal cavity, and the most common new metastatic sites identified at pathological autopsy were the esophagus, biliary system, stomach, pancreas, kidney, adrenal glands, heart and pericardium, colon, lung, and bone marrow. The fact that gastrointestinal examination was not performed in the absence of symptoms during the patient’s clinical courses may have contributed to the difficulty in detecting metastases to the gastrointestinal tract before death. Although the parenchymal organs were easily identified by imaging, metastases to the kidneys and adrenal glands were most common in the terminal stages of the disease, and a high percentage of these metastases were first revealed at pathological autopsy. Cardiac and pericardial metastases are relatively rare [15], and these sites are difficult to examine without symptoms, such as cardiac tamponade. In the present study, these metastases were seen in four cases, three of which were first seen at pathological autopsy.

Our hypothesis in this study was that imaging closer to the date of death would contribute to a lower major discordance rate, but no association was found. A possible reason for this finding was that the major discrepancies were due to histological types, sepsis, or bone marrow disease that were not evident on imaging. The reported causes of death not associated with the antemortem clinical diagnosis include pulmonary infarction, sepsis, disseminated intravascular coagulation, and bone marrow disease [16]. While Ai has solid utility, it cannot replace all pathological autopsies. The present study was not performed using Ai. Although it cannot be definitively stated because Ai was not used in this study, no association was found between the proximity of the date of death and the date of imaging examination and the discordance rate, suggesting that imaging examination alone is insufficient to elucidate all pathological conditions.

PE is a reported pathophysiological cause of death in cancer patients that is not identified by antemortem imaging [17]. Abnormal coagulation capacity in cancer patients is well known, and fatal PE was found in approximately 10% of pathological autopsies of cancer patients in previous studies [18,19]. Of these autopsies, neoplastic PE was found in 14%. Despite the high prevalence of neoplastic PE at pathological autopsy, it is rarely diagnosed before death [20]. Only 10% of clinically suspected cases were diagnosed by antemortem imaging [21]. He et al. reported no significant difference in detection rates before and after 2005; therefore, pathological autopsy was still considered the “gold standard” for the diagnosis of neoplastic PE [21]. In the present study, three of 25 patients (12.0%) had pulmonary infarction, which is similar to the rates in previous reports [18-21]. Three patients had thrombotic pulmonary infarction with no tumor emboli, and three patients were not diagnosed with pulmonary infarction before death. This may be because respiratory deterioration due to cardiac failure, general weakness from peritonitis, and pneumonia in the terminal stage of the patient’s illnesses prevented a strong suspicion of pulmonary infarction, and the terminal stage of the illnesses prevented aggressive imaging.

This study has several limitations. First, it involved a small sample size and was conducted at a single institution within a single department. Second, variations in carcinoma types and treatment courses made it challenging to present a unified perspective. Third, as a retrospective study, inconsistencies in the timing and scope of post-mortem examinations complicated direct comparisons between antemortem findings and pathological autopsy results. Despite these limitations, this study highlights the presence of discrepancies between antemortem clinical diagnoses and pathological autopsy findings. These discrepancies underscore the importance of conducting pathological autopsies, with potential benefits for improving future clinical practice.

## Conclusions

Despite advances in the diagnosis and treatment of patients with malignant tumors, there is a significant discrepancy between clinical and pathological autopsy diagnoses. Metastases to the gastrointestinal tract, biliary system, and pancreas are frequently revealed during pathological autopsy. In some cases, sepsis and pulmonary infarction leading to death are revealed during pathological autopsy, even if the clinical diagnosis fails to identify these conditions. Pathological autopsy is useful to understand a patient’s pathological condition, as it reveals significant discrepancies between antemortem diagnoses and autopsy findings, particularly regarding disease extent, histological diagnosis, and causes of death.
